# Prevalence of childhood Cancer among children attending referral hospitals of outpatient Department in Ethiopia

**DOI:** 10.1186/s12885-021-08014-0

**Published:** 2021-03-12

**Authors:** Aklilu Endalamaw, Nega Tezera Assimamaw, Tadesse Awoke Ayele, Achenef Asmamaw Muche, Ejigu Gebeye Zeleke, Amare Wondim, Getaneh Mulualem Belay, Yeneabat Birhanu, Ashenafi Tazebew, Masresha Asmare Techane, Selam Fisha Kassa, Chalachew Adugna Wubneh

**Affiliations:** 1 Department of Pediatrics and Child Health Nursing, School of Health Sciences, College of Medicine and Health Sciences, Bahir Dar University, Bahir Dar, Ethiopia; 2 Department of Pediatrics and Child Health Nursing, School of Nursing, College of Medicine and Health Sciences, University of Gondar, P.O.BOX 196, Gondar, Ethiopia; 3 Department of Epidemiology and Biostatistics, Institute of Public Health, College of Medicine and Health Sciences, University of Gondar, Gondar, Ethiopia; 4 Department of Surgical Nursing, School of Nursing, College of Medicine and Health Sciences, University of Gondar, Gondar, Ethiopia; 5 Department of Pediatrics and Child Health, School Medicine, College of Medicine and Health Sciences, University of Gondar, Gondar, Ethiopia

**Keywords:** Cancer, Children, Ethiopia

## Abstract

**Introduction:**

Childhood cancer is one of the leading causes of morbidity and mortality in the pediatrics age group. The problem affects both developed and developing countries. A high mortality rate has been observed in low-income counties. Despite its high fatality rate, less attention has been paid to the problem in developing countries, including Ethiopia. For this reason, childhood cancer is not well documented in the study setting. Therefore, we assessed the prevalence of childhood cancer in Ethiopia.

**Methods:**

Institution based cross-sectional study design from January 1, 2019, to March 30, 2019, was conducted in the pediatrics treatment center. A systematic random sampling technique has used to select 1270 children in the pediatric outpatient department. The data were entered using Epi info version 7 and exported to SPSS version 20 for analysis. We checked model fitness for the advanced statistical methods, but it was difficult to proceed with logistic regression model to see the association between dependent and explanatory variables because of the unmet x^2^ assumption. We presented the results by using tables and figures.

**Results:**

From the total 1270 study participants, 1257 were included in the final analysis provided that a 98.97% response rate. Out of these, 10(0.8%) children were diagnosed with cancer. Regarding its types, two each, Acute Lymphocytic Leukemia, Wilms tumor, Hodgkin lymphoma, and one each non-Hodgkin lymphoma, Parotid cancer, Retinoblastoma, and Breast cancer were reported. The prevalence of childhood cancer was 0.9 and 0.7% among male and female children, respectively.

**Conclusions:**

Eight children diagnosed with cancer per 1000 children who visited the pediatric outpatient department. Even though childhood cancers have little attention from policymakers, the prevalence of childhood cancer remains prevalent. Therefore, researchers and policymakers shall give special emphasis to childhood cancer.

## Introduction

Cancer is a collective term that refers to the body’s uncontrolled abnormal cell growth and reproduction. This abnormal cell could destroy healthy tissue and then, spread beyond its usual boundaries [1]. Non-communicable diseases (NCD) including cancer considered as the disease of “adult only” in a sense children do not get cancer and childhood cancer not cured, which is a noticeable common false perception of the community but it is a preventable cause of morbidity, disability, and mortality [2–4]. Over 90% of childhood cancer deaths occur in low-resource settings because many children in low-and middle-income countries do not receive or complete care [5].

The burden of childhood cancer has been reported both in developed and developing countries. According to 2015 World Health Organization (WHO) report each year, more than 200,000 children are diagnosed with cancer globally. It is projected that an estimated 21 million people will be diagnosed by 2030 [6, 7]. It is estimated that 80–85% of pediatric cancer cases occur in the developing world but 10% of them have survived [8]. In the USA, it is reported that there are an estimated 15,780 children between the ages of birth and 19 years of age who are diagnosed with cancer. Approximately 1 in 285 children diagnosed with cancer before their 20th birthday in the USA [9] and 174 cases per million children per year in Australia [10]. In Yemen, the incidence of childhood cancer is 1.9 per 100,000 and this disease was more common in different age groups of 5–9 years (35%), 10–14 years (33.7%), and 0–4 years (31%) [11]. Similarly, cancer is an increasing public health burden for Ethiopia and Sub-Saharan Africa at large [12]. According to hospital records published in 2015 in Ethiopia, there are more than 150,000 cancer cases per year approximately 6000 new cases of pediatric cancer each year [13]. Different strategies have been implemented to prevent specific cancer-causing agents. Notably, cancer caused by the human papillomavirus and hepatitis B virus can be prevented through vaccination [14]. Furthermore, children who were exposed to radiation, like atomic bomb blast had a risk of leukemia, thyroid cancer risk due to a nuclear power plant, ultrasound, and computed tomography scan exposure during early pregnancy time. Family history of cancer, smoking history of the family, passive smoking exposure during pregnancy, white phosphorous, an overdose of radiation are some of the risk factors related to pediatric cancer [15–19]. On the other hand, inherent risk factors including birth weight, parental age, and congenital anomalies are consistently associated with most types of pediatric cancer which make challenging for pediatric cancer [20].

The extent of the cancer burden in the young population is unknown in many low-and middle-income countries. Even in the presence of population-based cancer registries, the collection of information about childhood cancers is often neglected and the least prioritized health issue similarly in Ethiopia.

Therefore, this study aimed to determine the prevalence of childhood cancer in Ethiopia. Hence, this study can be used as baseline data for health policymakers which help to design strategy in the country, childhood cancer researchers, and clinicians.

## Methods and materials

### Study design and period

An institution-based cross-sectional study design was conducted from January 1, 2019, to March 30, 2019.

### Study setting

The study was conducted at the University of Gondar Comprehensive Specialized Hospital, Gondar and Felege Hiwot Referral Hospital, Bahir Dar Ethiopia. Gondar city is about 750 km (km) far from the Northwest of Addis Ababa, the capital city of Ethiopia. Felege Hiwot referral hospital is found in Bahir Dar which is the city of Amhara region which is found around 321 km far from Addis Ababa. Both hospitals are serving more than 5 million people in their catchment areas.

### Source and study population

All children who attended the pediatrics outpatient department (OPD) in the University of Gondar Comprehensive Specialized Hospital and Felege Hiwot Referral Hospital were the source population. All children who visited the pediatrics’ OPD during the study period were considered as the study population.

### Sample size and sampling technique

The sample size was determined by using a single population proportion formula with the statistical assumptions of 95% Confidence Interval (CI), 50% proportion, and 5% Margin of error.

$$ \mathrm{n}=\frac{\left(\mathrm{Za}/2\right)2\times \left(1-\mathrm{P}\right)}{\left(\mathrm{W}\right)2} $$

Where,

$$ \mathrm{n}=\mathrm{initial}\ \mathrm{sample}\ \mathrm{size} $$

$$ \mathrm{Z}=1.96,\mathrm{the}\ \mathrm{corresponding}\ \mathrm{Z}-\mathrm{score}\ \mathrm{for}\ \mathrm{the}\ 95\%\mathrm{CI} $$

$$ \mathrm{P}=\mathrm{Proportion}=50\% $$

$$ \mathrm{W}=\mathrm{Margin}\ \mathrm{of}\ \mathrm{error}=5\%=0.05 $$

$$ \mathrm{n}=\frac{(1.96)2\times 0.5\left(1-0.5\right)}{(0.05)2}=384 $$

By considering a 10% non-response rate and 1.5 design effect, the final sample size for each site was 635. Finally, 1270 study participants were selected by using a systematic random sampling technique.

**Fig. 1:**
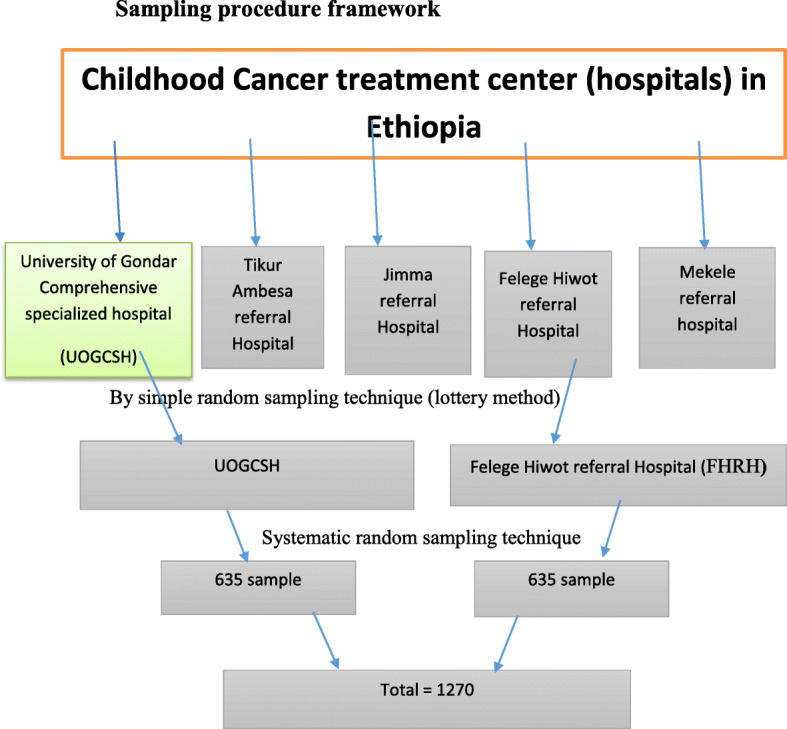
Sample selection procedure

From the University of Gondar Comprehensive Specialized Hospital, 40 children have visited the outpatient department every day on average 40 children/day = 1200/month = 3600/3 months. Every day, 8 study participants’ information was collected. K^th^ = Total visited per day/sample size per day = 40/8 = 5. Therefore, the study participant was selected every 5th child from the daily visited children. Similarly, in Felege Hiwot referral hospital, on average 36 children visit the hospital daily = 1080/month = 3240/3 month, 32/8 = 4. Therefore, every 4th patient was selected from the daily visited children until the required sample size reached (Fig. 1).

### Data collection tools and procedures

Data regarding the socio-demographic characteristics of children and their caregivers and related characteristics were collected through face-to-face interviews of the caregivers/children using a structured questionnaire adapted from different literature. The data were collected by 4 BSc nurses and strictly followed by 2 supervisors to the overall data collection process from two study sites. A one-day training was given to the data collectors and supervisors about the purpose of the study, data collection tools, collection techniques, and ethical issues during the selection of study participants and collection of the data. All answers to closed-and open-ended questions were written manually by the interviewer. The supervisors were assessing the consistency and completeness of data on daily basis.

For those children suspected of cancer was sent to pathology, laboratory, and imagining investigations for screening. Different diagnostic procedures were done according to the standard operation procedures of each suspected cases including Complete Blood Count and Peripheral Morphology examination, urine test, ophthalmic examination, ultrasound, magnetic resonance imaging, X-ray on the affected site, and biopsy and/or aspiration from the diseased gland or tissue based on the type and site of the cancer suspected. Their chart numbers, phone number, and follow-up time was taken and followed to make sure the final diagnosis status. Based on the aforementioned information, their pathology, laboratory, and imagining results were confirmed and collected.

### Study variables

#### Dependent variable

Prevalence of childhood cancer.

#### Independent variables

Socio-demographic characteristics of children and the caregivers, clinical presentation related characteristics of the children and, feeding and lifestyle-related characteristics.

### Statistical analysis

The data entry was performed using the statistical program Epi-info version 7 and then, exported into SPSS version 20 for analysis. Descriptive statistics were carried out and presented with narration, tabulation, and graphical presentation. We checked model fitness for the advanced statistical methods, but difficult to proceed with logistical regression model to see the association between dependent and explanatory variables because of the unmet x^2^ assumption and model fitness.

### Quality assurance mechanisms

To assure the quality of the data, the tool was prepared first in English and then, translated to the national language (Amharic) by language experts in English and Amharic. Data collectors and supervisors were trained on the data collection process for 1 day. The pretest was conducted from 5% of the total sample size in hospitals which is not selected for actual data collection. Appropriate modifications were made to the tool accordingly. Data collection was closely monitored by investigators and supervisors. Moreover, the data quality was assured by using statistical parameters for assessing the validity of the collected data.

### Ethical considerations

Ethical clearance was issued by the University of Gondar institutional ethical review board. Throughout the data collection, any harm was avoided to the study participants. Respecting the dignity of the study participant had been prioritized. The voluntary participation of respondents was considered. Moreover, participants had the right to withdraw from the study at any stage if they wish to do so. For all study participants, informed consent was obtained from the parent/legal guardian. In addition, assent also obtained from children as appropriate according to their age. In this study, all methods were performed in accordance with the Declaration of Helsinki.

## Results

### Sociodemographic characteristics of children and caregiver

From the total 1270 study participants, 1257 mother-child pairs were included in the final analysis provided that a 98.97% response rate. The minimum and maximum age of children was 1 month and 179 months with the mean of 32.4 (SD = 46.2) months, respectively. The minimum and maximum age of the caregiver was 16 years and 61 years with the mean of 32.4 (SD = 6.8) years, respectively. The minimum and maximum income of caregivers per month was 0.0 Ethiopian Birr (ETB) and 9000 ETB with the mean of 2022.7 ETB (SD = 1994.3), respectively. Regarding the marital status of the caregiver, most (93%) were married and 71% were urban residence. Female caregivers were the dominant (82.2%) and more than half (56.4%) were male children (Table 1).

**Table 1:** Socio-demographic-related variables of children and caregivers attending clinic at FHRH and UOGCSH, Northwest Ethiopia in 2019

Variables	Frequency (***n*** = 1257)	Percentage (%)
**Age of the child in year**		
< 1 year	320	25.5
1–5 year	567	45.1
5–10 year	214	17
≥ 10 year	156	12.4
**Sex of the child**		
Male	709	56.4
Female	548	43.6
**Residence of the child**		
Urban	892	71.0
Rural	365	29.0
**Age of caregiver in years**		
15–25 year	165	13.1
26–35 year	708	56.3
36–45 year	331	26.3
> 45 year	53	4.2
**Sex of care giver**		
Male	224	17.8
Female	1033	82.2
**Region of caregiver**		
Amhara	1216	96.7
Tigray	11	0.9
Oromia	4	0.3
Others	26	2.1
**Religion of caregiver**		
Orthodox	1126	89.6
Muslim	118	9.4
Protestant	11	0.9
Others	2	0.2
**Marital status of caregiver**		
Single	57	4.5
Married	1169	93.0
Others	31	2.5
**Educational status of care giver**		
Illiterate	211	16.8
Primary school	395	31.4
Secondary school	272	21.6
College/University	379	30.2
**Occupation of the caregiver**		
Not employed	221	17.6
Government employed	341	27.1
Farmer	141	11.2
Merchant	176	14.0
Daily Laborer	39	3.1
Housewife	339	27.0
**Monthly income (ETB)**		
No regular monthly income	304	24.2
Less than 1000	252	20
1000-3000	366	29.1
3001–5000	260	20.7
5001-10,000	75	6.0

### Feeding and lifestyle-related characteristics of children’s families

Almost all (98.2%) of the children are from non-smoker family and 61.3% did not frequently consume hot foods and drinks. Most (93.4%) of the families did not frequently eat red raw meat. About 75.9 and 75.5% of the family members did not frequently consume fabricated food and not utilized herbicides/pesticides, respectively (Table 2).

**Table 2:** Feeding and lifestyle of related-variables of children’s families attend clinic at FHRH and UOGCSH, Northwest Ethiopia in 2019

Variables	Frequency (***n*** = 1257)	Percentage (%)
**Smoker family member**		
No	1235	98.2
Yes	22	1.8
**Frequent consumption of hot foods and drinks**		
No	771	61.3
Yes	486	48.7
**Frequent consumption of red raw meat**		
No	1174	93.4
Yes	83	6.6
**Frequent consumption of fabricated food**		
No	954	75.9
Yes	303	24.1
**Using herbicides or pesticides**		
No	949	75.5
Yes	308	24.5

### Clinical presentation of the children

From the total children included in this study, 28.24% presented in the hospital with chief complain of cough or difficulty of breathing followed by fever (13.44%) and diarrhea (10.82%) (Table 3).

**Table 3:** Children’s clinical characteristics attending clinic at FHRH and UOGCSH, Northwest Ethiopia in 2019

Variables	Frequency (***n*** = 1257)	Percentage (%)
**Chief complain (*n*** = 1257)*		
Vomiting	71	5.65
Diarrhea	136	10.82
Fever	169	13.44
Cough/Difficulty of breathing	355	28.24
Abdominal distention (pain)	68	5.41
Swelling	40	3.18
Itching/rash	41	3,26
Ear problem/pain/discharge	14	1.11
Difficulty of swallowing	19	1.51
Loss of appetite	17	1.35
Chest pain	11	0.875
Fatigue	28	2.23
Eye problem	64	5.09
Pain/burning sensation during urination	21	1.67
Other**	213	16.95

*A child may have more than one chief complain, ** those chief complains having less than 11 frequency were merged as others

### Prevalence of cancer among children

**Fig. 2:**
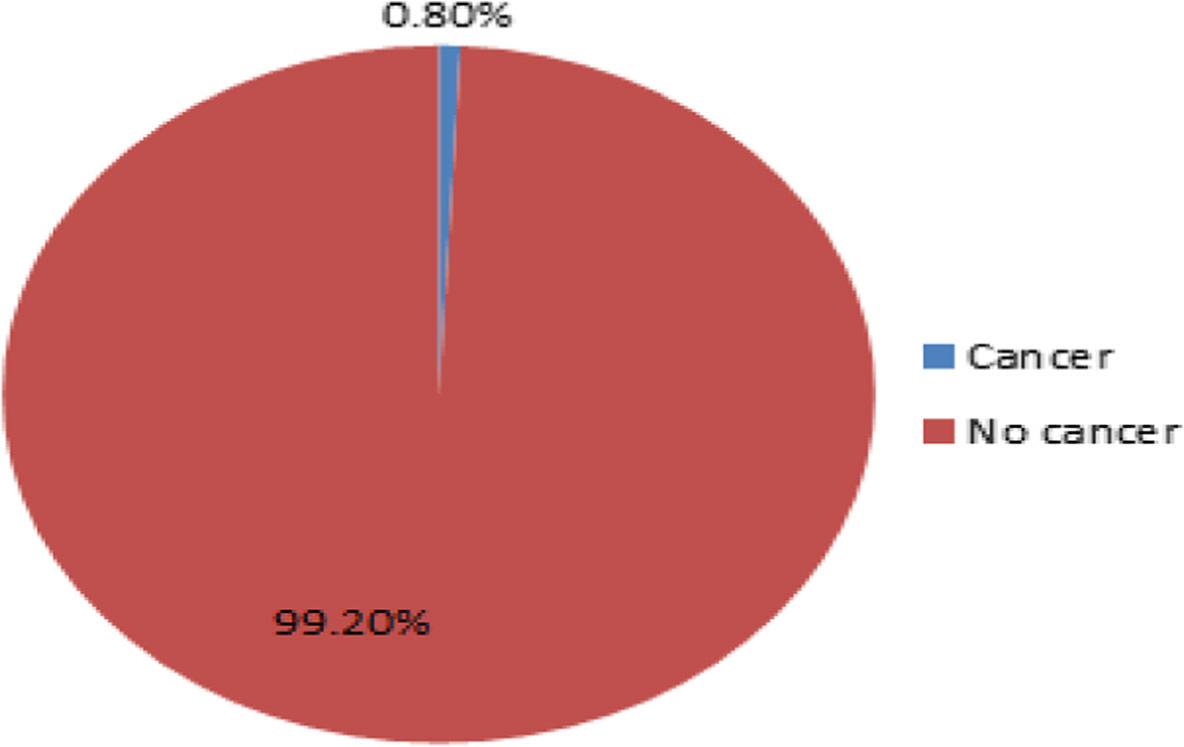
Pie chart presentation of cancer prevalence

From the total 1257 mother-child pair participants, 32(2.5%) children screened for cancer. Regarding the cancer status of the child, 10(0.8%) children were diagnosed with different types of childhood cancer (Fig. 2). Two Acute Lymphocytic Leukemia (ALL), two Wilms tumor, two Hodgkin lymphoma, one non-Hodgkin lymphoma, one Parotid cancer, one Retinoblastoma, and one male Breast cancer were reported in the study period from the two study settings (Figure-2).

### Distribution of cancer across variables

The prevalence of childhood cancer was 0.9 and 0.7% among male and female children, respectively. Six out of 10 were from urban residence families and regarding parents’ occupation, 3 from merchant and 4 were from a farmer family. Six of them were in the age group from1–5 years. All cancer positive children were from married caregivers (Table 4).

**Table 4:** Prevalence of childhood cancer among children attending clinic at FHRH and UOGCSH, Northwest Ethiopia in 2019

Variables	Cross-tabulation (***n*** = 1257)	
	Cancer	No cancer
**Sex of the child**		
Male	5	704
Female	5	543
**Residence**		
Urban	6	886
Rural	4	361
**Age of the child**		
Less than 1 year	1	319
1–5 year	6	561
5–10 year	1	213
10 year and above	2	154
**Age of the care giver**		
15–25 year	0	165
26–35 year	5	703
36–45 year	4	327
Above 45 year	1	52
**Sex of the caregiver**		
Male	3	221
Female	7	1026
**Educational status of care giver**		
Illiterate	3	208
Primary school	5	390
Secondary school	2	270
College/University	0	379
**Occupation**		
Not employed	0	221
Government employed	1	340
Farmer	4	137
Merchant	3	173
Daily Laborer	1	38
Housewife	1	338
**Monthly income (ETB)**		
No regular monthly income	0	304
Less than 1000	4	248
1000-3000	6	360
3001–5000	0	260
5001-10,000	0	75
**Religion of caregiver**		
Orthodox	9	1117
Muslim	1	117
Protestant	0	11
Others	0	2

## Discussion

Despite an increasing burden, childhood cancer was poorly quantified and continues to receive low public health priority in Ethiopia, largely because of the overwhelming burden of communicable diseases and limited resources. Hence, we aimed to determine the prevalence of childhood cancer and predictors among mother-child pairs in two referral hospitals in Ethiopia. In this study, the prevalence of childhood cancer was 0.8% (95%, CI: 0.3–1.3). This finding is a bit lower than studies conducted in Africa that ranged between 1.4% in Ghana to 10.0% in Rwanda [21], and 1.6 to 4.8% in India [22]. The discrepancy might be due to differences in the diagnostic capacity of the health institutions or availability of diagnostic facilities, health-seeking behavior/norms of the population, and perhaps access to health services. For instance, only 23% of health facilities offer services for cancer in Ethiopia [23]. Accurate epidemiological data on pediatric cancers will enable the mobilization of sufficient resources for proper screening, prevention, and treatment of cancers. Most of the children with cancer in our analysis resided in the urban area of the study setting due to the proximity and its accessibility of medical resources. But a child from a rural area might have died before registration or diagnosis as access to health care is very deficient. Hence, improved diagnostics and suspicion on the clinical examination might result in an increasing number of patients diagnosed with cancer in rural areas. The WHO global initiative for childhood cancer aimed to increase the overall survival for six key childhood cancers (acute lymphoblastic leukemia, Burkitt’s lymphoma, Hodgkin lymphoma, low-grade glioma, retinoblastoma, and Wilms tumor) to 60% globally by 2030 through the development of national centers of excellence and expanding regional satellites [24]. Moreover, in low- and middle-income countries, the survival of children is highly dependent on several health indicators, including the number of physicians and nurses per 1000 population, infrastructure, human resources, and level of supportive care [25]. However, from more than 46 hospitals in Amhara regional state only two centers were found who had pediatric-specific cancer center which makes very difficult in the accessibility of early diagnosis and treatment. This will have ended with poor survival for children with childhood cancer in resource- limited countries like Ethiopia.

In this study, males were more affected than female children. The finding was supported with previous studies, risk factors for childhood cancer including demographic, environmental, intrinsic, and genetic factors [20, 22, 26]. On sociodemographic factors for most childhood cancers, there is a slight male preponderance as reported in the previous studies [13, 20, 22] but equal distribution was observed in this study. Again, in this study, more than half of the child cancer were occurred in the age group from 1 to 5 years in a similar manner from the childhood cancer age groups, from 0 to 4-years age group had the greatest contribution in Australia [27] and to global childhood cancer analysis [24].

Concerning distribution, two ALL, two Wilms tumor, two Hodgkin lymphomas were reported in this study. Similarly, the most common childhood cancer is leukemia in India [22] and Australia [27]. In other studies, leukemia and retinoblastomas in Namibia [28], leukemia and lymphomas in central Sudan [29], Burkitt lymphoma, retinoblastoma, and Wilms tumor in Northwest Cameroon [30] were reported. In Western Africa, Non-Hodgkin lymphoma was the most common in Ghana, in Ivory Coast and Mali [21]. Furthermore, in eastern Africa, Uganda recorded Kaposi sarcoma (KS) as the most common tumor in children while two Kenyan centers reported mainly Burkitt lymphoma (BL) [31] and lymphomas, Wilms tumor, and retinoblastoma in North-West Ethiopia [32]. The difference would be due to variation in genetic makeup and environmental risk factor.

Children with cancer in Sub-Saharan Africa face paramount challenges as children with cancer in low-income countries often present with advanced-stage disease, lack of health care facilities, transportation, information about the disease, and treatment options [33]. In a similar manner in Ethiopia, apart from this finding, a previous study also revealed that children with cancer were exposed to prolonged hospitalization due to a lack of anti-cancer drugs, adequate diagnostic and treatment facilities [13]. As strength the study figures out the epidemiology of childhood cancer in the study setting. The other strength of this study, it will serve as baseline for further study on the area and informative for health works and other stockholders in the area. As limitation the study was institution based among children who visit the OPD for different illness which may not representative for the children who is apparently healthy and may have cancer in the community. The limitation was due feasibility issue in terms of resource.

## Conclusions

Although childhood cancers receive little attention from local policymakers, the prevalence of childhood cancer remains prevalent in the study setting. Meanwhile, an increased number of reported cases might occur with the increase in awareness, knowledge, diagnostic tools, and affordability. Therefore, large-scale community-based study shall be conducted to address those children who did not gate the access.

## Abbreviation

ALL: Acute Lymphocytic Leukemia
ETB: Ethiopian Birr
FHRH: Felege Hiwot Referral Hospital
Km: Kilometers
NCD: Non-communicable Diseases
OPD: Outpatient Department
UOGCSH: University of Gondar Comprehensive Specialized Hospital
USA: United State of America
WHO: World Health Organization
